# Cigarette smoking, cadmium exposure, and zinc intake on obstructive lung disorder

**DOI:** 10.1186/1465-9921-11-53

**Published:** 2010-05-09

**Authors:** Yu-Sheng Lin, James L Caffrey, Man-Huei Chang, Nicole Dowling, Jou-Wei Lin

**Affiliations:** 1 Department of Environmental and Occupational Health, University of North Texas Health Science Center, Fort Worth, TX 76107, USA; 2 Department of Integrative Physiology and Cardiovascular Research Institute, University of North Texas Health Science Center, Fort Worth, TX 76107, USA; 3 National Office of Public Health Genomics, Centers for Disease Control and Prevention, 1600 Clifton Rd., NE MS: E-61, Atlanta, GA 30333, USA; 4 Cardiovascular Center and Health Management Center, National Taiwan University Hospital Yun-Lin Branch, Dou-Liou City, Taiwan; 5 Department of Medicine, College of Medicine, National Taiwan University, Taipei, Taiwan

## Abstract

**Background and objective:**

This study examined whether zinc intake was associated with lower risk of smoking-induced obstructive lung disorder through interplay with cadmium, one of major toxicants in cigarette smoke.

**Methods:**

Data were obtained from a sample of 6,726 subjects aged 40+ from the Third National Health and Nutrition Examination Survey. The forced expiratory volume in 1 second (FEV1) and forced vital capacity (FVC) were measured using spirometry. Gender-, ethnicity-, and age-specific equations were used to calculate the lower limit of normal (LLN) to define obstructive lung disorder as: observed FEV1/FVC ratio and FEV1 below respective LLN. Zinc intake was assessed by questionnaire. Logistic regression analysis was applied to investigate the associations of interest.

**Results:**

The analyses showed that an increased prevalence of obstructive lung disorder was observed among individuals with low zinc intake regardless of smoking status. The adjusted odds of lung disorder are approximately 1.9 times greater for subjects in the lowest zinc-intake tertile than those in the highest tertile (odds ratio = 1.89, 95% confidence interval = 1.22-2.93). The effect of smoking on lung function decreased considerably after adjusting for urinary cadmium. Protective association between the zinc-to-cadmium ratio (log-transformed) and respiratory risk suggests that zinc may play a role in smoking-associated lung disorder by modifying the influence of cadmium.

**Conclusions:**

While zinc intake is associated with lower risk of obstructive lung disorder, the role of smoking cession and/or prevention are likely to be more important given their far greater effect on respiratory risk. Future research is warranted to explore the mechanisms by which zinc could modify smoking-associated lung disease.

## Background

Obstructive lung disorders including chronic obstructive pulmonary disease (COPD) are characterized by chronic airway inflammation and ensuing airflow limitation. Although cigarette smoking is the most important risk factor for obstructive lung disease, the underlying mechanisms are still not completely understood. For instance, it has been suggested that COPD results from smoking-associated inflammation and oxidative damage to key enzymes (e.g., alpha 1-antitrypsin deficiency) [1], but not all smokers develop COPD [2], and some former smokers have persistent inflammation and remain at risk [3].

Both animal and human epidemiologic data indicate that exposure to cadmium (Cd), a constituent of cigarette smoke, is associated with oxidative stress and chronic inflammation [4-7]. Increasing evidence indicates that Cd may play a role in smoking-induced disorders including impaired lung function [8,9], diabetes and hypertension [10-12]. Of note, once entering the body, Cd is transported in the blood and bound to plasma proteins, mainly metallothionein [13], a cysteine rich protein and scavenger of OH radical [14]. Thus the binding of metallothionein with Cd is an essential adaptation to Cd poisoning because it can prevent free Cd ions from exerting their toxicity [15]. The resulting metallothionein mediated reduction in Cd toxicity however, potentially comes at the expense of lowering the reserve capacity for buffering OH radicals; thus exposing tissues to oxyradical damage from other sources [16].

Given zinc (Zn) is a trace element and an effective inducer of metallothionein, we proposed the hypothesis that the risk of developing smoking-associated obstructive lung disease could be modified by Zn intake through modification of Cd toxicity. To test this hypothesis, we evaluated whether the association between obstructive lung disorders and cigarette smoking varies with dietary Zn intake, accounting for other risk factors. The analysis was conducted using a population-based, nationally representative sample from the Third National Health and Nutrition Examination Survey (NHANES III, 1988-94).

## Materials and methods

### Data source and study population

The NHANES is a series of national health examination surveys, conducted by the National Center for Health Statistics (NCHS) of the Center for Disease Control and Prevention, to collect data on the health and nutritional status of a representative sample of the non-institutionalized civilian US population by using a multistage, stratified sampling design [17]. The protocol was approved by the NCHS Research Ethics Review Board (ERB) and all subjects provided written informed consent. The analysis of this study was restricted to 8,745 non-Hispanic Whites, non-Hispanic Blacks, and Mexican Americans aged 40 yrs or older with valid spirometry measurements in the NHANES III survey (1988-94). A total of 868 subjects with missing information for covariates of interest (e.g., urinary Cd) were excluded from the analyses. Pregnant subjects (n = 65) and those with unreliable or incomplete information on Zn intake, such as type, frequency, and amount of vitamin and mineral supplement use (n = 1,086) were excluded. This resulted in a final sample of 6,726 subjects in the current analysis.

### Lung function measurement and Definition of obstructive lung disorders

Lung function was assessed with standard determinations of the forced expiratory volume in 1 second (FEV1) and forced vital capacity (FVC) with dry rolling-seal spirometer (Ohio 827 rolling seal spirometer; Ohio Medical Instrument Company, Cincinnati, Ohio) following the procedures described by the American Thoracic Society (ATS) in 1987 [18]. The largest FVC and FEV1 obtained from acceptable maneuvers were used for the analysis. As suggested by ATS and European Respiratory Society (ERS) [19], the predicted values for the lower limit of normal (LLN) of the FEV1/FVC ratio and FEV1 were calculated for each subject using gender-, ethnicity-, and age-specific equations reported by Hankinson et al. (1999) [20]. In the current analysis, the subject was categorized as having obstructive lung disorder if his/her observed FEV1/FVC ratio and FEV1 were less than respective LLN [20,21].

### Collection of demographic, dietary, and laboratory data

Self-reported demographic characteristics including age, gender, body mass index (BMI), race/ethnicity, and cigarette smoking status were obtained during the survey interview. BMI was calculated from measured height and weight, and categorized as underweight (<18.5 kg/m^2^), normal (18.5-24.9 kg/m^2^), overweight (25.0-29.9 kg/m^2^), and obese (≥ 30 kg/m^2^) [22]. Smoking status was classified as: never-smokers (<100 life time cigarettes), former smoker (> = 100 but not currently smoking), and current smoker (> = 100 and current smoking) [23]. The average time of smoking cession for former smokers was 18.2 years (range: <1 - 87 years)(data not shown). Pack-years of smoking was also determined as the reported average number of packs smoked per day by the number of years smoked (1 pack-year = 20 cigarettes/day for 1 year). The concentrations of serum cotinine (the metabolite of nicotine) were also measured from all subjects using high performance liquid chromatography (HPLC)-atmospheric pressure chemical ionization tandem mass spectrometry [24] to control for environmental smoking.

Dietary intake data were collected by trained interviewers using an automated NHANES III Dietary Data Collection System described previously [25]. In brief, total intake of daily Zn was estimated by summing dietary Zn intake from food, beverage and supplements (vitamins and mineral products used during the past month) assessed by 24-hr dietary recall and food-frequency interviews. Urinary Cd concentration, commonly used to characterize cadmium exposure [26,27], was measured using Zeeman graphite furnace atomic absorption spectrometry (Perkin-Elmer Corp., Norwalk, CT) [24] with a detection limit of 0.01 μg/L and was adjusted for urinary creatinine [28,29].

### Statistical analyses

To investigate the role of Zn intake in smoking-related lung disorder, the differences in prevalence of obstructive lung disorder across demographic characteristics were first assessed with Cochran-Mantel-Haenszel chi-square tests. The odds ratios (OR) with 95% confidence interval (95% CI) generated from logistic regression models were then used to examine the association of interests. The interactions of zinc intake with smoking and cadmium exposure were also examined, and the chi-squared trend test was used to evaluate whether there were trends in the odds ratios across categories of zinc intake and cigarette smoking/cadmium exposure. Logarithmic transformations were performed to normalize the data distribution where necessary. We applied mobile examination center (MEC)/home-examined statistical sampling weight to account for the complex stratified multistage sampling in NHANES III and used SUDAAN 9.03 (Research Triangle Institute, 2004) with the Taylor series linearization method [30] to obtain unbiased standard errors for all statistical analyses. Thus, the percentages and regression estimates reported here represent estimates for the U.S. population. Tertile cutoffs of dietary Zn and urinary Cd were determined according to the weighted distribution in the study samples. The level of statistical significance was set at 0.05.

## Results

The crude prevalence of obstructive lung function by demographic characteristics of the participants aged 40 years or older is shown in table 1. As expected, the prevalence of obstructive lung disorder increased in the order: never-smokers (2.99%) < former smokers (9.55%) < active smokers (17.7%). A similar pattern of results was evident for pack-years of cigarettes as: zero pack-years (3.08%) < greater than zero-19 pack years (6.50%) < greater than 20 pack-years (19.6%). Individuals with low Zn intake (in the low tertile of Zn intake < 8.35 mg/day) had higher prevalence of obstructive lung disorder than did those with middle (8.35-14.4 mg/day) and high (> 14.4 mg/day) Zn intake (*p *= 0.01). The geometric mean and 5th-to-95th percentile range for daily Zn intake were 11.0 and 4.09-34.4 mg/day, respectively (data not shown). Also, obstructive lung disorder was generally more prevalent among elderly and non-Hispanic whites. For instance, the prevalence of individuals with obstructive lung function among those aged 55 years or older was approximately twice that of subjects aged 40-54. BMI was inversely associated with obstructive lung function that was significantly increased among underweight subjects (BMI less than 18.5) as compared to subjects with higher BMI. The relationship of obstructive lung disorder with low body weight may represent existing poor health in this subgroup. Overall, the age-adjusted (2000 U.S. population) prevalence of obstructive lung disease among U.S. adults aged 40+ was 8.63% (95% CI = 7.39-10.1%, data not shown).

**Table 1:** Prevalence of obstructive lung disorder by demographic characteristics

Characteristics	N	Prevalence of obstructive lung function (95%CI)^a^	*P*-value^b^
Smoking status			< 0.001
Never-smokers	2948	2.99 (2.14-4.16)	
Former smokers	2306	9.55 (7.77-11.7)	
Active smokers	1472	17.7 (15.2-20.6)	
Pack years of cigarettes			< 0.001
0	3171	3.08 (2.24-4.23)	
>0-19	1874	6.50 (5.05-8.33)	
≥ 20	1681	19.6 (16.7-22.9)	
Zinc intake, mg/d			0.01
Tertile 1 (< 8.35)	2464	11.4 (8.78-14.7)	
Tertile 2 (8.30-14.4)	2135	8.66 (6.99-10.7)	
Tertile 3 (>14.4)	2127	6.60 (5.39-8.07)	
Age, yrs			< 0.001
40-54	2484	5.97 (4.38-8.10)	
55 or older	4242	11.5 (10.2-12.9)	
Gender			0.16
Male	3288	9.55 (8.06-11.3)	
Female	3438	8.13 (6.62-9.94)	
Race/Ethnicity			< 0.001
Mexican American	1522	4.86 (3.70-6.37)	
Non-Hispanic black	1500	5.58 (4.09-7.57)	
Non-Hispanic white	3704	9.26 (7.93-10.8)	
Body mass index, kg/m^2^			0.001
<18.5	106	33.9 (20.0-51.3)	
18.5-24.9	2120	9.80 (7.66-12.5)	
25-29	2634	7.96 (6.59-9.60)	
≥ 30	1866	7.10 (5.87-8.57)	

^a ^Obstructive lung disorder was defined as: observed FEV1/FVC ratio < [FEV1/FVC]_LLN _and observed FEV1 < [FEV1]_LLN _[20,21]. The estimated prevalence of obstructive lung disorder was calculated using the NHANES III sample weights.

^b ^Cochran-Mantel-Haenszel chi-square test.

Abbreviations: FEV1, forced expiratory volume in 1 second; FVC, forced volume vital capacity; LLN, lower limit of normal.

When all of the covariates were considered jointly in a multiple logistic regression model on obstructive lung disease, the protective effect of Zn intake remained significant (table 2). For instance, as shown in model 1, those who currently had lowest Zn intake were twice as likely to have obstructive lung disorder compared to those with the highest tertile of Zn intake after adjustment for covariates (OR = 1.97, 95% CI = 1.28-3.03). Of other risk factors examined in the study, cigarette smoking is the leading cause of obstructive lung disorder, followed by BMI, age, and race-ethnicity. Comparable results were obtained by replacing smoking status with pack years of cigarettes (Additional file 1).

**Table 2:** Multivariate-adjusted logistic regression of obstructive lung disorder using smoking status as the measure of tobacco exposure

	Model 1^b^		Model 2^b^	
		
	OR (95% CI)	*p*	OR (95% CI)	*p*
Smoking status		< 0.001		< 0.001
Never-smokers	1.00 (1.00-1.00)		1.00 (1.00-1.00)	
Former smokers	3.37 (2.21-5.14)		2.60 (1.67-4.06)	
Active smokers	7.66 (4.97-11.79)		4.38 (2.71-7.08)	
Zinc intake, mg/d		0.01		0.01
Tertile 1 (< 8.35)	1.97 (1.28-3.03)		1.89 (1.22-2.93)	
Tertile 2 (8.30-14.4)	1.36 (0.95-1.96)		1.29 (0.91-1.82)	
Tertile 3 (>14.4)	1.00 (1.00-1.00)		1.00 (1.00-1.00)	
Age, yrs (55 or older)	2.39 (1.75-3.25)	< 0.001	1.82 (1.33-2.49)	< 0.001
Gender (male)	1.13 (0.84-1.53)	0.38	1.37 (0.99-1.89)	0.05
Race/Ethnicity		< 0.001		0.001
Mexican American	0.61 (0.42-0.88)		0.61 (0.41-0.89)	
Non-Hispanic black	0.45 (0.30-0.69)		0.47 (0.31-0.73)	
Non-Hispanic white	1.00 (1.00-1.00)		1.00 (1.00-1.00)	
Body mass index, kg/m^2^		0.001		0.001
<18.5	3.99 (1.81-8.79)		3.63 (1.72-7.63)	
18.5-24.9	1.00 (1.00-1.00)		1.00 (1.00-1.00)	
25-29	0.83 (0.62-1.11)		0.81 (0.60-1.11)	
≥ 30	0.78 (0.56-1.08)		0.79 (0.56-1.11)	
Urinary cadmium, μg/g creatinine				0.001
Tertile 1 (< 0.39)	-		1.00 (1.00-1.00)	
Tertile 2 (0.39-0.79)	-		1.54 (0.98-2.43)	
Tertile 3 (>0.79)	-		3.48 (2.54-4.76)	

^a ^Obstructive lung disorder was defined as: observed FEV1/FVC ratio < [FEV1/FVC]_LLN _and observed FEV1 < [FEV1]_LLN _[20,21]. The estimated prevalence of obstructive lung disorder was calculated using the NHANES III sample weights.

^b ^Both model 1 and 2 accommodated smoking status, zinc intake, and other covariates including age, gender, race/ethnicity, and body mass index, whereas model 2 was further adjusted for urinary cadmium.

Abbreviations: FEV1, forced expiratory volume in 1 second; FVC, forced volume vital capacity; LLN, lower limit of normal.

**Figure 1:**
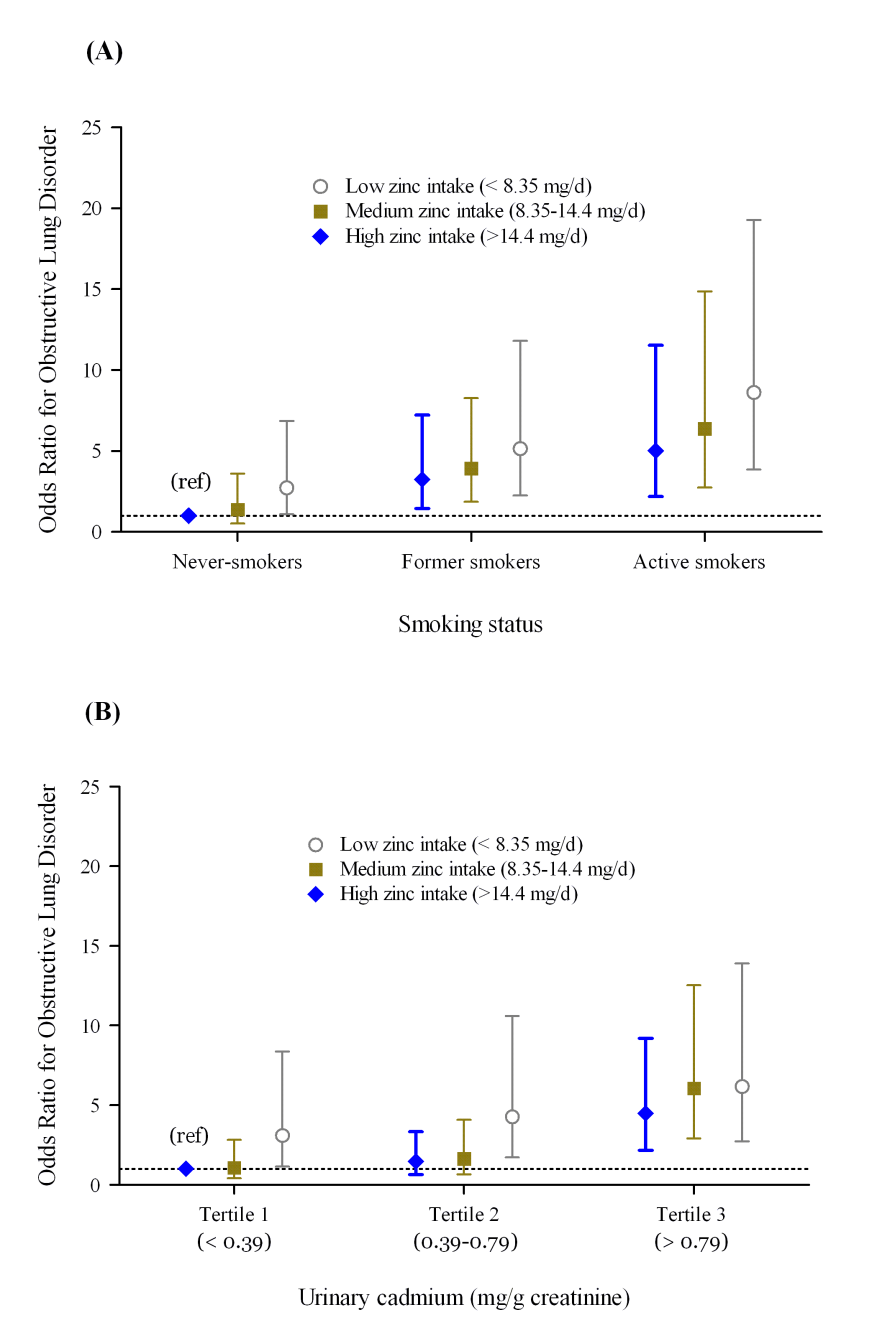
**Adjusted odds ratios for obstructive lung disorder by (A) smoking status and daily zinc intake (adjusted for age, body mass index, gender, race/ethnicity, and urinary cadmium); (B) urinary cadmium and daily zinc intake (adjusted for age, body mass index, gender, race/ethnicity, and smoking status)**.

**Figure 2:**
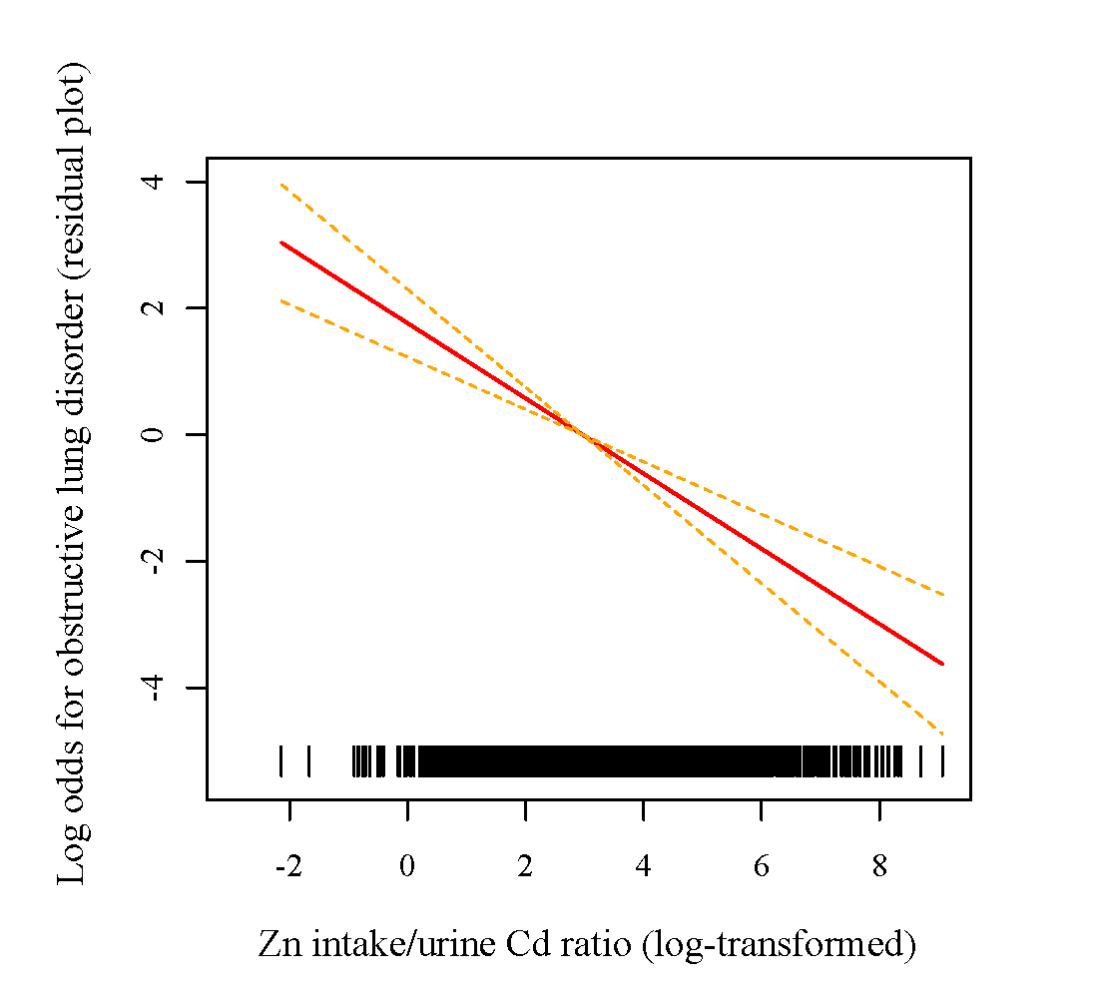
**Log-odds of obstructive lung disorder versus the ratio of Zn to Cd (log-transformed) (adjusted for age, body mass index, gender, race/ethnicity, and smoking status)**. Dotted lines: twice-standard-error; rug plot on the x-axis describing the distribution of the ratio of Zn to Cd (log-transformed).

Interestingly, the effect (estimated OR) of smoking on the obstructive lung disorder decreased approximately 20-40% in model 2 as compared to model 1. Whereas the influence of Zn on respiratory risk remained the same, the odds ratios for both smoking status and urinary Cd were reduced by another 15-50% with further adjustment of pack years of cigarettes (data not shown). Indeed, pack years of cigarettes, urinary Cd, and smoking status were significantly correlated with each other. For instance, urinary Cd was associated with both pack years of cigarettes (Spearman correlation = 0.34, *p *< 0.001, data not shown) and cigarette smoking status that the geometric means (standard error) for urinary Cd were 0.87 (0.04), 0.53 (0.02), and 0.36 (0.02) μg/g creatinine in active smokers, former smokers, and never-smokers, respectively (*p *< 0.001, data not shown). On the other hand, the cadmium effect on obstructive lung disorder is also significant and is independent of smoking (model 2). Despite the lack of statistical significance for either Zn-smoking (*p *= 0.68) or Zn-Cd interactions (*p *= 0.06) (data not shown), there are positive trends in the odds ratios among individuals who had low Zn intake across all smoking status categories (figure 1a), or urinary cadmium concentrations (figure 1b) (*P*_*trend *_< 0.05 for both). In addition, there was an inverse relationship between Zn intake and urinary Cd following adjustment for other covariates (the estimated regression coefficient ± standard error = -0.097 ± 0.023, *p *< 0.001, data not shown). The plot of an adjusted log-odds of obstructive lung disorder versus the ratio of Zn to Cd suggested that higher ratios were in fact protective (figure 2). These results indicate that Zn may moderate the toxic role of Cd in cigarette smoking.

## Discussion

While the current findings are consistent with earlier studies suggesting that smoking is the leading cause of obstructive lung disease in the U.S. population aged 40+ [31,32], we also found that Zn intake is associated with lower risk of obstructive lung disorder across cigarette smoking status. The effect persisted even after adjustment for other respiratory risk factors such as age. Of note, the negative effect of cigarette smoking on obstructive lung disorder decreased after adjusting for urinary Cd in the multivariable analyses. Indeed, cigarette smoking is one of the major sources of environmental exposure to Cd [33,34]. It has been suggested that Cd plays an important role in promoting oxidative stress and inflammation [5,6]. The current findings support the prior evidence suggesting that cadmium exposure, as a risk factor independent of cigarette smoking, is associated with impaired lung functions and presumably other cadmium-associated diseases such as cardiovascular disease [8,35]. Considering its 10-30 year half-life in the body compared to other shorter lived constituents of cigarette smoke [13,36], Cd might reasonably explain some of the sustained obstructive lung disorder observed among former smokers who had not smoked in years [3].

The current analyses reveal a protective association between the zinc-to-cadmium ratio (log-transformed) and reduced respiratory risk suggesting that Zn may moderate the risk of smoking-associated lung disorders through interplay with Cd. Zn is essential to the production of metallothionein, a key component in the detoxification kinetics of Cd in the human body [13,14]. Metallothionein alone can passively extract the interfering Cd and reduce its toxic load directly. The inverse association between Zn and Cd adds support to the hypothesis that when sufficient Zn is available, metallothionein not only extracts the offending Cd, but actively restores structure and function by donating the missing Zn [37]. Alternatively, the positive influence of Zn may also result from the anti-inflammatory and anti-oxidant activities of a spectrum of Zn-dependent enzymes and transcription factors [38,39]. It was found, for instance, reported that zinc can decrease the production of inflammatory cytokines such as tumor necrosis factor-α (TNF-α) and interleukin-1β (IL-1β) via inhibition of NF-kappaB activation [38].

The current findings were generally compatible with the U.S. Recommended Dietary Allowances (RDAs) for Zn intake at 11 and 8 mg/day for men and women aged 19+, respectively [40]. Adequate intake of Zn is associated with lower risk of obstructive lung disease, presumably through mitigation of inflammatory and oxidative stresses associated Cd exposure. A potential benefit of higher Zn intake may exist for former smokers, who demonstrate a consistent trend toward lower risk at higher Zn intakes suggesting again that the Zn-Cd exchange may be an important clearance mechanism. Although the most appropriate Zn intake related to a lower risk of obstructive lung disorder is unclear, the increased respiratory risk of low Zn intake is apparent even in never smokers.

There were several limitations of the current study that need to be addressed. First, the cross-sectional design of NHANES data only permits the investigation of associations rather than causation among Cd, Zn, smoking, and obstructive lung disease. The results, nevertheless, were biologically plausible and supported by epidemiologic and animal data [15,38]. Future work with a longitudinal follow-up design would help verify these findings. Second, despite its demonstrated validity as a reliable measurement of food intake [41,42], the 24-hour recall and self-reported dietary supplement data may not provide a precise estimation of Zn intake. When available, serum or urinary Zn levels, which were not measured in NHANES III, could help clarify the role of Zn in smoking-associated lung disease. Finally, although we adjusted for a number of confounding factors, such as age, the confounding influences of unmeasured factors (e.g. genetic background) cannot be excluded. For instance, tumor necrosis factor-alpha (TNF-α) and interleukin-10 (IL-10) represent pro- and anti-inflammatory cytokines, respectively, and polymorphisms in TNF-α and IL-10 have been associated with obstructive lung disease [43,44], a complex disease characterized by airway obstruction and inflammation. Thus, a delicate balance between pro- and anti-inflammatory responses could well determine protection from or susceptibility to the pathogenesis of obstructive lung disease such as COPD [45].

In conclusion, the current study demonstrated that Zn intake is associated with lower a risk of developing smoking-associated obstructive lung disorder for smokers and non-smokers alike. The interplay between Zn and Cd presumably plays a role in mediating the toxic effect of smoking. Although Zn intake is associated with lower risk of obstructive lung disease, the risk reduction associated with smoking cessation or never smoking is much greater. Thus, smoking prevention and cessation programs should remain a cornerstone of public health policy to reduce the subsequent risk of obstructive lung disease.

## Selected Abbreviations

Cd: Cadmium; COPD: Chronic obstructive pulmonary disease; LLN: Lower limit of normal; NHANES III: The Third National Health and Nutrition Examination Survey; OR: Odds ratio; Zn: zinc; 95% CI: 95% confidence interval.

## Competing interests

The authors declare that they have no competing interests.

## Authors' contributions

JWL had full access to all of the data in the study and takes responsibility for the integrity of the data and the accuracy of the data analysis. YSL carried out the study. JWL and MHC participated in the design of the study and performed the statistical analysis. JLC and ND participated in the data interpretation and drafted the manuscript. All authors read and approved the final manuscript.

## Disclaimers

The findings and conclusions in this report are those of the author(s) and do not necessarily represent the views of the Centers for Disease Control and Prevention. The corresponding author has full access to all of the data in the study and takes responsibility for the integrity of the data and the accuracy of the data analysis. The authors do not have any affiliation with NHANES.

## Supplementary Material

Additional file 1 Multivariate-adjusted logistic regression of obstructive lung disorder using pack-years of cigarettes as the measure of tobacco exposure. Click here for file

## References

[B1] Macnee W Pathogenesis of chronic obstructive pulmonary disease Clin Chest Med 2007 28 479 513 v 10.1016/j.ccm.2007.06.008 17720039

[B2] Lundback B Lindberg A Lindstrom M Ronmark E Jonsson AC Jonsson E Larsson LG Andersson S Sandstrom T Larsson K Not 15 but 50% of smokers develop COPD?--Report from the Obstructive Lung Disease in Northern Sweden Studies Respir Med 2003 97 115 122 10.1053/rmed.2003.1446 12587960

[B3] Sutherland ER Martin RJ Airway inflammation in chronic obstructive pulmonary disease: comparisons with asthma J Allergy Clin Immunol 2003 112 819 827 quiz 828 10.1016/S0091-6749(03)02011-6 14610463

[B4] Kataranovski M Kataranovski D Savic D Jovcic G Bogdanovic Z Jovanovic T Granulocyte and plasma cytokine activity in acute cadmium intoxication in rats Physiol Res 1998 47 453 461 10453753

[B5] Kirschvink N Martin N Fievez L Smith N Marlin D Gustin P Airway inflammation in cadmium-exposed rats is associated with pulmonary oxidative stress and emphysema Free Radic Res 2006 40 241 250 10.1080/10715760500494657 16484040

[B6] Lin YS Rathod D Ho WC Caffrey JJ Cadmium Exposure Is Associated With Elevated Blood C-Reactive Protein and Fibrinogen in the U. S. Population: The Third National Health and Nutrition Examination Survey (NHANES III, 1988-1994) Ann Epidemiol 2009 19 592 596 10.1016/j.annepidem.2009.02.005 19406663

[B7] Kundu S Sengupta S Chatterjee S Mitra S Bhattacharyya A Cadmium induces lung inflammation independent of lung cell proliferation: a molecular approach J Inflamm (Lond) 2009 6 19 10.1186/1476-9255-6-19 19523218 PMC2702298

[B8] Mannino DM Holguin F Greves HM Savage-Brown A Stock AL Jones RL Urinary cadmium levels predict lower lung function in current and former smokers: data from the Third National Health and Nutrition Examination Survey Thorax 2004 59 194 198 10.1136/thorax.2003.012054 14985551 PMC1746977

[B9] Lampe BJ Park SK Robins T Mukherjee B Litonjua AA Amarasiriwardena C Weisskopf M Sparrow D Hu H Association between 24-hour urinary cadmium and pulmonary function among community-exposed men: the VA Normative Aging Study Environ Health Perspect 2008 116 1226 1230 10.1289/ehp.11265 18795167 PMC2535626

[B10] Schwartz GG Il'yasova D Ivanova A Urinary cadmium, impaired fasting glucose, and diabetes in the NHANES III Diabetes Care 2003 26 468 470 10.2337/diacare.26.2.468 12547882

[B11] Everett CJ Frithsen IL Association of urinary cadmium and myocardial infarction Environ Res 2008 106 284 286 10.1016/j.envres.2007.10.009 18053980

[B12] Tellez-Plaza M Navas-Acien A Crainiceanu CM Guallar E Cadmium exposure and hypertension in the 1999-2004 National Health and Nutrition Examination Survey (NHANES) Environ Health Perspect 2008 116 51 56 10.1289/ehp.10764 18197299 PMC2199293

[B13] Casarett LJ Klaassen CD Watkins JB Casarett and Doull's essentials of toxicology 2003 New York: McGraw-Hill/Medical Pub. Div 822 827

[B14] Prasad AS Bao B Beck FW Kucuk O Sarkar FH Antioxidant effect of zinc in humans Free Radic Biol Med 2004 37 1182 1190 10.1016/j.freeradbiomed.2004.07.007 15451058

[B15] Ohta H Seki Y Imamiya S Metallothionein-like cadmium binding protein in rat testes administered with cadmium and selenium Bulletin of Environmental Contamination and Toxicology 1988 41 195 200 10.1007/BF01705430 3207901

[B16] Maret W Krezel A Cellular zinc and redox buffering capacity of metallothionein/thionein in health and disease Mol Med 2007 13 371 375 10.2119/2007-00036.Maret 17622324 PMC1952669

[B17] Centers for Disease Control and Prevention(CDC). National Center for Health Statistics (NCHS) Plan and operation of the Third National Health and Nutrition Examination Survey, 1988-94. Series 1: programs and collection procedures Vital Health Stat 1 1994 1 407 7975354

[B18] American Thoracic Society Standardization of spirometry--1987 update Am Rev Respir Dis 1987 136 1285 1298 3674589 10.1164/ajrccm/136.5.1285

[B19] Pellegrino R Viegi G Brusasco V Crapo RO Burgos F Casaburi R Coates A Grinten CPM van der Gustafsson P Hankinson J Interpretative strategies for lung function tests Eur Respir J 2005 26 948 968 10.1183/09031936.05.00035205 16264058

[B20] Hankinson JL Odencrantz JR Fedan KB Spirometric Reference Values from a Sample of the General U.S. Population Am J Respir Crit Care Med 1999 159 179 187 9872837 10.1164/ajrccm.159.1.9712108

[B21] Jiang R Paik DC Hankinson JL Barr RG Cured meat consumption, lung function, and chronic obstructive pulmonary disease among United States adults Am J Respir Crit Care Med 2007 175 798 804 10.1164/rccm.200607-969OC 17255565 PMC1899290

[B22] Expert Panel on the Identification E Treatment of Overweight and Obesity in Adults Executive Summary of the Clinical Guidelines on the Identification, Evaluation, and Treatment of Overweight and Obesity in Adults Arch Intern Med 1998 1855 1867 10.1001/archinte.158.17.1855 9759681

[B23] Cigarette Smoking-Attributable Morbidity--United States, 2000 JAMA 2003 290 1987 1988 10.1001/jama.290.15.1987 12966360

[B24] Gunter EW Lewis BL Koncikowski SM Laboratory Methods used for the Third National Health and Nutrition Examination Survey (NHANES III), 1988-1994 Included in CD-ROM 6-0178 NHANES III Reference Manuals and Reports 1996 Hyattsville, MD: Centers for Disease Control and Prevention

[B25] Briefel RR Bialostosky K Kennedy-Stephenson J McDowell MA Ervin RB Wright JD Zinc intake of the U.S. population: findings from the third National Health and Nutrition Examination Survey, 1988-1994 J Nutr 2000 130 1367S 1373S 10801945 10.1093/jn/130.5.1367S

[B26] Choudhury H Harvey T Thayer WC Lockwood TF Stiteler WM Goodrum PE Hassett JM Diamond GL Urinary cadmium elimination as a biomarker of exposure for evaluating a cadmium dietary exposure--biokinetics model J Toxicol Environ Health A 2001 63 321 350 10.1080/15287390152103643 11471865

[B27] Jin T Nordberg G Ye T Bo M Wang H Zhu G Kong Q Bernard A Osteoporosis and renal dysfunction in a general population exposed to cadmium in China Environ Res 2004 96 353 359 10.1016/j.envres.2004.02.012 15364604

[B28] Pruszkowska E Carnrick GR Slavin W Direct determination of cadmium in urine with use of a stabilized temperature platform furnace and Zeeman background correction Clin Chem 1983 29 477 480 6825258

[B29] Mason HJ Williams NR Morgan MG Stevenson AJ Armitage S Influence of biological and analytical variation on urine measurements for monitoring exposure to cadmium Occup Environ Med 1998 55 132 137 10.1136/oem.55.2.132 9614399 PMC1757554

[B30] Centers for Disease Control and Prevention (CDC) National Center for Health Statistics (NCHS) Analytic and Reporting Guidelines: The Third National Health and Nutrition Examination Survey, NHANES III (1988-94) 1996 Hyattsville, MD: U.S. Department of Health and Human Services, Centers for Disease Control and Prevention http://www.cdc.gov/nchs/data/nhanes/nhanes3/nh3gui.pdf [accessed 7 January 2009]

[B31] Hogg JC Chu F Utokaparch S Woods R Elliott WM Buzatu L Cherniack RM Rogers RM Sciurba FC Coxson HO Pare PD The nature of small-airway obstruction in chronic obstructive pulmonary disease N Engl J Med 2004 350 2645 2653 10.1056/NEJMoa032158 15215480

[B32] Raherison C Girodet P-O Epidemiology of COPD EUROPEAN RESPIRATORY REVIEW 2009 18 213 221 10.1183/09059180.00003609 20956146

[B33] Noonan CW Sarasua SM Campagna D Kathman SJ Lybarger JA Mueller PW Effects of exposure to low levels of environmental cadmium on renal biomarkers Environ Health Perspect 2002 110 151 155 11836143 10.1289/ehp.02110151 PMC1240729

[B34] Satarug S Moore MR Adverse health effects of chronic exposure to low-level cadmium in foodstuffs and cigarette smoke Environ Health Perspect 2004 112 1099 1103 15238284 10.1289/ehp.6751 PMC1247384

[B35] Trzcinka-Ochocka M Jakubowski M Razniewska G Halatek T Gazewski A The effects of environmental cadmium exposure on kidney function: the possible influence of age Environ Res 2004 95 143 150 10.1016/j.envres.2003.10.003 15147919

[B36] Hecht SS Human urinary carcinogen metabolites: biomarkers for investigating tobacco and cancer Carcinogenesis 2002 23 907 922 10.1093/carcin/23.6.907 12082012

[B37] Roesijadi G Metal transfer as a mechanism for metallothionein-mediated metal detoxification Cell Mol Biol (Noisy-le-grand) 2000 46 393 405 10774928

[B38] Prasad AS Clinical, immunological, anti-inflammatory and antioxidant roles of zinc Exp Gerontol 2008 43 370 377 10.1016/j.exger.2007.10.013 18054190

[B39] Shenkin A Trace elements and inflammatory response: implications for nutritional support Nutrition 1995 11 100 105 7749254

[B40] Institute of Medicine (U.S.). Panel on Micronutrients DRI: dietary reference intakes for vitamin A, vitamin K, arsenic, boron, chromium, copper, iodine, iron, manganese, molybdenum, nickel, silicon, vanadium, and zinc: a report of the Panel on Micronutrients. and the Standing Committee on the Scientific Evaluation of Dietary Reference Intakes, Food and Nutrition Board, Institute of Medicine 2001 Washington, D.C.: National Academy Press

[B41] Hu FB Rimm E Smith-Warner SA Feskanich D Stampfer MJ Ascherio A Sampson L Willett WC Reproducibility and validity of dietary patterns assessed with a food-frequency questionnaire Am J Clin Nutr 1999 69 243 249 9989687 10.1093/ajcn/69.2.243

[B42] Byers T Food frequency dietary assessment: how bad is good enough? Am J Epidemiol 2001 154 1087 1088 10.1093/aje/154.12.1087 11744510

[B43] Verweij CL Tumour necrosis factor gene polymorphisms as severity markers in rheumatoid arthritis Ann Rheum Dis 1999 58 Suppl 1 I20 26 10.1136/ard.58.2008.i20 10577969 PMC1766583

[B44] Hackett TL Holloway R Holgate ST Warner JA Dynamics of pro-inflammatory and anti-inflammatory cytokine release during acute inflammation in chronic obstructive pulmonary disease: an ex vivo study Respir Res 2008 9 47 10.1186/1465-9921-9-47 18510721 PMC2435536

[B45] Dentener MA Creutzberg EC Schols AM Mantovani A van't Veer C Buurman WA Wouters EF Systemic anti-inflammatory mediators in COPD: increase in soluble interleukin 1 receptor II during treatment of exacerbations Thorax 2001 56 721 726 10.1136/thorax.56.9.721 11514694 PMC1746133

