# Supplementary material for: Cigarette smoking, cadmium exposure, and zinc intake on obstructive lung disorder
Source: Respir Res. 2010 May 9;11(1):53. doi: 10.1186/1465-9921-11-53 (PMC2881897; doi:10.1186/1465-9921-11-53)
Supplement: Additional file 1 — Multivariate-adjusted logistic regression of obstructive lung disorder using pack-years of cigarettes as the measure of tobacco exposure. [file 1465-9921-11-53-S1.DOC]

|  | **Additional file 1. Multivariate-adjusted logistic regression of obstructive lung disorder using pack-years of cigarettes as the measure of tobacco exposure *^a^*** | | | | | | |  |
| --- | --- | --- | --- | --- | --- | --- | --- | --- |
|  |  |  | Model 1*^b^* | |  | Model 2*^b^* | |  |
|  |  |  | OR (95% CI) | *p* |  | OR (95% CI) | *p* |  |
|  | Pack years of cigarettes |  |  | < 0.001 |  |  | < 0.001 |  |
|  | ≥ 20 |  | 1.00 (1.00-1.00) |  |  | 1.00 (1.00-1.00) |  |  |
|  | >0-19 |  | 2.34 (1.54-3.57) |  |  | 1.87 (1.22-2.86) |  |  |
|  | 0 |  | 7.87 (5.16-12.0) |  |  | 4.96 (3.06-8.04) |  |  |
|  | Zinc intake, mg/d |  |  | 0.002 |  |  | 0.01 |  |
|  | Tertile 1 (< 8.35) |  | 1.99 (1.34-2.96) |  |  | 1.90 (1.25-2.90) |  |  |
|  | Tertile 2 (8.30-14.4) |  | 1.35 (0.93-1.94) |  |  | 1.27 (0.90-1.80) |  |  |
|  | Tertile 3 (>14.4) |  | 1.00 (1.00-1.00) |  |  | 1.00 (1.00-1.00) |  |  |
|  | Age, yrs (55 or older) |  | 1.78 (1.30-2.43) | < 0.001 |  | 1.51 (1.10-2.07) | 0.01 |  |
|  | Gender (male) |  | 0.96 (0.70-1.32) | 0.78 |  | 1.19 (0.84-1.68) | 0.32 |  |
|  | Race/Ethnicity |  |  | 0.06 |  |  | 0.05 |  |
|  | Mexican American |  | 0.76 (0.52-1.09) |  |  | 0.73 (0.50-1.06) |  |  |
|  | Non-Hispanic black |  | 0.66 (0.46-0.96) |  |  | 0.65 (0.44-0.95) |  |  |
|  | Non-Hispanic white |  | 1.00 (1.00-1.00) |  |  | 1.00 (1.00-1.00) |  |  |
|  | Body mass index, kg/m^2^ |  |  | < 0.001 |  |  | < 0.001 |  |
|  | <18.5 |  | 4.36 (1.96-9.66) |  |  | 3.82 (1.81-8.08) |  |  |
|  | 18.5-24.9 |  | 1.00 (1.00-1.00) |  |  | 1.00 (1.00-1.00) |  |  |
|  | 25-29 |  | 0.70 (0.53-0.93) |  |  | 0.72 (0.53-0.97) |  |  |
|  | ≥ 30 |  | 0.64 (0.47-0.86) |  |  | 0.69 (0.50-0.95) |  |  |
|  | Urinary cadmium, µg/g creatinine | |  |  |  |  | < 0.001 |  |
|  | Tertile 1 (< 0.39) |  | - |  |  | 1.00 (1.00-1.00) |  |  |
|  | Tertile 2 (0.39-0.79) |  | - |  |  | 1.39 (0.87-2.23) |  |  |
|  | Tertile 3 (>0.79) |  | - |  |  | 3.02 (2.14-4.26) |  |  |
|  | ^a^ Obstructive lung disorder was defined as: observed FEV1/FVC ratio < [FEV1/FVC]_LLN_  and observed FEV1 < [FEV1]_LLN_ [20-21]. The estimated prevalence of obstructive lung disorder was calculated using the NHANES III sample weights.  ^b^ Both model 1 and 2 accommodated pack-years of cigarettes, zinc intake, and other covariates including age, gender, race/ethnicity, and body mass index, whereas model 2 was further adjusted for urinary cadmium.  Abbreviations: FEV1, forced expiratory volume in 1 second; FVC, forced volume vital capacity; LLN, lower limit of normal. | | | | | | |  |
